# Cancer of the Larynx

**Published:** 1908-02-01

**Authors:** 


### Laryngology and Rhinology.

### CANCER OF THE LARYNX.

In our last article we described the type of epithelioma of the vocal cord which shows itself as a single polypoid outgrowth; the two other principal forms of the disease in this region are a diffuse infiltration and a congestion or hyperæmia of one cord. In the former the cord is converted into a mass of nodular or papillary infiltration; the margin is irregular and often presents a peculiar fringe-like appearance. The nodular variety of tuberculous laryngitis may exactly resemble this form of epithelioma, and there is no doubt that tuberculosis of this type has frequently been diagnosed as cancer, especially when it occurs, as is by no means uncommon, in elderly men; in such cases there is often no suspicion of phthisis, and the physical signs in the lungs may be very uncertain or may appear to indicate only a simple bronchitis, while tubercle bacilli in the sputum may be very scanty. If the infiltration affect both cords, the diagnosis becomes simpler or there may be some slight infiltration of the arytenoid or interarytenoid region to arouse a suspicion of tuberculous laryngitis; but when such an infiltration occurs in an elderly individual who shows no sign of being consumptive, it is very natural to conclude at once that the disease is cancerous. More rarely syphilitic infiltration shows a similar appearance, but the voice is more raucous, the hoarseness of longer duration and other parts of the larynx are usually affected as well; a syphilitic ulcer on the cord has a sharply-cut margin surrounded by a zone of hyperæmia and a smooth and shiny base. Some cases of pachydermia laryngis, especially of the warty variety, may resemble epitheliomatous infiltration; they are, however, seldom unilateral, and the affection is very chronic, so that there will usually be a history of vocal impairment of many years' duration Sometimes, at its earliest beginning, the growth appears as a localised fusiform swelling of the cord, of a yellowish colour, and with enlarged vessels ramifying upon its surface. This appearance, which is not common, is very characteristic, especially when combined with impaired mobility of the cord.

Diffuse hyperæmia of the cord is sometimes, though more rarely, the first sign of the disease, and this should be remembered in cases of such congestion without apparent cause. There is usually in addition some swelling of the cord, and the appearance is probably due to the fact that the actual growth is hidden from sight beneath the cord. A unilateral congestion of a vocal cord should always arouse attention, for, if there be a marked difference between the two sides it is seldom the result of simple inflammation; the effect can be produced by syphilis, tubercle, or traumatism as well as by epithelioma. Here the sign of impaired mobility is again of value, for a unilateral congestion, combined with a lagging movement of the hyperæmic cord, is decidedly suggestive of malignant disease. Gouty laryngitis occasionally produces a unilateral redness and even, in rare cases, a diffuse granular infiltration of the cord, which may closely imitate epithelioma; in gouty laryngitis, however, the symptoms will usually be more acute, with greater soreness and irritability, and accompanied by dusky congestion of the fauces, pharynx, or other parts of the larynx.

Epitheliomata of the cord spread very slowly at first, but later they grow more rapidly and extend beyond the limits of the cord upwards on to the ventricular band, and especially backwards to the posterior commissure, and so reach the opposite side. As a rarity, inoculation of the opposite cord by contact has been described.

Elsewhere, as on the ventricular bands, posterior commissure, or aryteno-epiglottidian folds, epithelioma usually manifests itself as a red infiltration with an irregular papillary surface. Such an epithelioma within the ventricle, or a spheroidal-cell cancer originating deeply in the mucous glands, may persist unnoticed for a considerable time; small papillary excrescences may appear on the surface, but after removal may show under the microscope no signs of malignancy because the base of the growth has not been included in the section.

Extrinsic growths extend rapidly to the pharynx and deep ulceration often occurs early; a favourite site, especially in women, is at the lower end of the pharynx on the back of the cricoid plate. The growth is then frequently out of sight, and manifests itself only by causing dysphagia; at the most, fixation and œdema of the arytenoid, or paralysis of a recurrent laryngeal nerve, can be made out by the laryngoscope. The tumour may sometimes be felt with the finger or by the œsophageal bougie. The diagnosis of these difficult cases may on occasions be cleared up by direct inspection with Killian's tube-speculum, but it is especially difficult to maintain the instrument in position in this region; a general anæsthetic is usually required and there is some risk in passing a metal speculum in such cases when it is impossible to tell beforehand how deeply the ulceration may extend. A better method has recently been introduced by von Eicken and called "hypopharyngoscopy" *; a stout probe is introduced into the anterior commissure, after thorough cocainisation, and the larynx is drawn strongly forwards while inspection is made with the laryngoscope mirror in the ordinary way.

If there is the least doubt of the diagnosis of a case of suspected epithelioma, a piece should always be removed for microscopical examination before any operation is undertaken. Certain cautions must, however be given in this connection. Firstly, this should never be done unless the case is considered operable and until the patient has given his consent to operation should the examination prove the disease to be cancer; for otherwise the procedure has no practical value, and the cutting irritates the tumour, and has often caused rapid growth and dissemination. In any case, too much reliance must not be placed on the microscopical appearance if it be opposed to the clinical aspect of the case.

* Harold Barwell on Hypopharyngoscopy. *Lancet*, 1907, vol. ii., p. 433.

